# Editorial: Entering the RNA Wonderland: Opportunities and Challenges for RNA Therapeutics in the Cardiovascular System

**DOI:** 10.3389/fphys.2020.00060

**Published:** 2020-02-07

**Authors:** Anna Zampetaki, Lars Maegdefessel

**Affiliations:** ^1^King's British Heart Foundation Centre, King's College London, London, United Kingdom; ^2^Department of Vascular and Endovascular Surgery, Klinikum Rechts der Isar, Technical University of Munich, Munich, Germany; ^3^German Center for Cardiovascular Research (DZHK), Munich, Germany; ^4^Department of Medicine, Karolinska Institutet, Stockholm, Sweden

**Keywords:** non-coding RNA, gene editing, RNA structure, cardiovascular diseases, RNA therapeutics

The discovery that transcription is pervasive with the vast majority of the genome encoding transcripts not translated into proteins, has transformed our understanding of the basic unit of genetic information (Ulitsky and Bartel, 2013). Non-coding RNAs (ncRNAs) display distinct expression profiles in different pathologies and have sparked interest for their potential role in tissue homeostasis and disease (Zampetaki and Mayr, 2012; Thum and Condorelli, 2015). This Research Topic describes the key bioinformatic and experimental tools to identify ncRNA expression and elucidate their mode of function and focuses on the recent findings on ncRNAs in cardiovascular diseases (CVDs) and the exciting developments in RNA therapeutics.

Advances in high-throughput sequencing platforms have facilitated the identification of ncRNAs. Technologies such as RNA sequencing (RNA-seq) are powerful tools for gene expression profiling and can sequence large numbers of DNA fragments in parallel producing millions of short reads in a single run (Metzker, 2010). These massive datasets are then processed in a number of computational steps to identify the transcripts in the RNA sample and provide an estimate of their abundance in the dataset. Weirick et al. review the computational tools that can be employed with a particular emphasis on datasets derived from endothelial cells. They discuss the methods to characterize lncRNAs that are not annotated and the bioinformatic programs to detect RNA editing within RNA-seq data.

Bioinformatic analysis of RNA-seq data also revealed a novel class of lncRNA transcripts, the circular RNAs (circRNAs) that emerge by RNA “backsplicing,” whereby the spliceosome fuses a splice donor site in a downstream exon to a splice acceptor site in an upstream exon (Memczak et al., 2013). They are produced in a cell-type specific manner, are stable against exonucleolytic decay and recent evidence suggests that they can exert biologically meaningful functions. Holdt et al. report on the latest findings on circRNA in CVDs, the potential therapeutic approaches based on either modulation of native circRNAs by therapeutic knockdown or by ectopic expression and the prospect of engineering non-native (artificial) circRNAs. The major hurdles for therapeutic strategies targeting circRNA in terms of design, delivery, and side effects are considered.

Once the transcripts are correctly annotated, understanding the molecular mechanism of action is a prerequisite for the development of RNA based therapeutic interventions. Intriguingly, lncRNAs demonstrate poor nucleotide sequence conservation (Hezroni et al., 2015). However, they tend to fold into thermodynamically stable secondary structures, such as double helixes and hairpins and conservation of the secondary structure rather than the sequence was proposed to define lncRNA function. Zampetaki et al. and Zampetaki et al. elaborate on the implications and challenges in linking function and lncRNA structure to design novel RNA therapeutic approaches and the experimental tools to determine the RNA structure.

LncRNAs exert their function through interaction with DNA, RNA and proteins to form ribonucleoprotein complexes (RNP). Their interaction with RNA binding proteins (RBPs) is thought to be crucial for very diverse cellular functions (Guttman and Rinn, 2012). High throughput screening techniques and *in silico* analysis have enabled us to interrogate protein-RNA binding, identify and predict binding motifs and sequence patterns in RBP-lncRNA interactions. These platforms can provide useful insights into RNP perturbations in disease. The review of Yang et al. reports on the RBP-regulated RNA networks in diabetes. Proper function of this intricate post-transcriptional RNA network is essential for the vascular endothelium and its disruption is associated with endothelial dysfunction under diabetic conditions. The clinical implication of their manipulation and the prospect of targeting RBPs or RBP-RNA interactions as a therapeutic strategy against diabetic vasculopathy are discussed in detail.

Apart from diabetic vasculopathy, ncRNAs have a profound effect in the vasculature at baseline and in disease. Hung et al. report on the distinct mechanisms of function for microRNAs (18–23 nt) and lncRNAs and the large body of evidence demonstrating their pleiotropic effects in pathological processes in vascular diseases. The potential of ncRNAs as effectors and biomarkers in vascular pathology is critically evaluated and insights into the technical limitations in establishing a standard protocol to ensure robust reproducibility for circulating ncRNAs as biomarkers in vascular diseases are provided.

In the heart, an increasing number of studies highlight the critical regulation of lncRNAs in cardiac disorders. Hobuß et al. summarize the function of lncRNAs in the development and progression of cardiac diseases with a particular emphasis on their molecular mode of action in pathological tissue remodeling. They also examine the challenges that have to be overcome to establish lncRNA based therapies and effective intervention strategies in the heart. In addition, the prognostic and diagnostic value of lncRNAs in biological fluids as a novel class of circulating biomarkers for heart diseases and the prospect of using these molecular fingerprints to replace protein-based indicators of disease is discussed.

Extending beyond ncRNA, RNA therapeutics focus on RNA as a prime target for therapeutic applications. Laina et al. highlight the two main designs to target RNA and modulate gene expression the double-stranded small interfering RNAs (siRNAs) and single stranded antisense oligonucleotides (ASOs) their advantages and limitations. The review also summarizes results from the clinical trials of RNA-targeting interventions and elaborates on the advances and hurdles for RNA based therapeutic applications. The future prospect of RNA therapeutics to empower precision medicine implementation and fulfill the promise of patient specific therapeutics is also evaluated.

In conclusion, this Research Topic elucidates the current understanding about the mechanisms of function of ncRNA and its role in CVDs and highlights the major advancements and promising developments in RNA therapeutics. Despite the significant progress this is a new field of research and several challenges remain. Better understanding of the mechanisms of function of ncRNAs and integration of innovative approaches to enhance target binding affinity, cellular uptake and efficient *in vivo* delivery of targeting agents are required to bring RNA based therapeutics closer to the clinic.

## Author Contributions

AZ and LM wrote and revised the manuscript.

### Conflict of Interest

The authors declare that the research was conducted in the absence of any commercial or financial relationships that could be construed as a potential conflict of interest.
